# Supplementary material for: Glucose Transporter 9 (GLUT9) Plays an Important Role in the Placental Uric Acid Transport System
Source: Cells. 2022 Feb 11;11(4):633. doi: 10.3390/cells11040633 (PMC8870656; doi:10.3390/cells11040633)
Supplement: Supplementary file 1 [file cells-11-00633-s001.zip › cells-1561723-supplementary.pdf]

cells

Supplementary Materials

Raising and Testing Anti-GLUT9a and -b Antibodies

To analyze the cellular and subcellular localization of GLUT9 isoforms via immunocytochemistry, we raised specific antibodies against GLUT9a and GLUT9b in rabbits using N-terminal peptides of the GLUT9a and GLUT9b proteins. Specificity was tested using a HEK-293 cell line overexpressing GLUT9a or GLUT9b as a positive control. Immunofluorescence staining revealed that both antibodies were highly specific and did not cross-react with the other isoform (Figure 1). The antibodies were further tested in human kidney tissue, which expresses high amounts of GLUT9. Immunocytochemistry showed GLUT9a to co-localize with the basal side of renal tubular cells, whereas GLUT9b was found to be expressed exclusively on apical membranes (data not shown). These findings are in line with previous studies [1]. Furthermore, GLUT9a-specific antibodies recognized GLUT9a in Western blots using membranes from human embryonic kidney cells transfected with GLUT9a or GLUT9b and fused to a C-terminal FLAG tag as a control (data not shown).

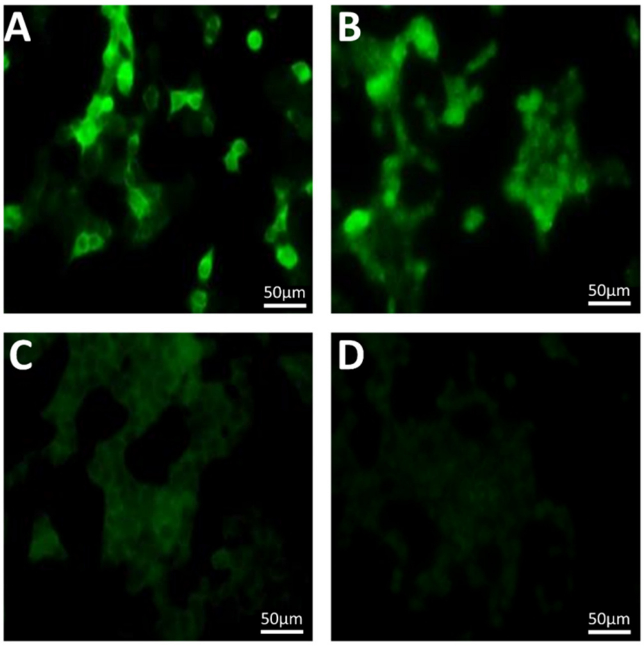

Supplementary Figure S1. Test of GLUT9-specific antibodies using transfected HEK-293 cells. (**A**) HEK-293 cells overexpressing hGLUT9a stained with a rabbit anti-hGLUT9a antibody. Scale bar represents 50 µm. (**B**) HEK-293 cells overexpressing hGLUT9b stained with a rabbit anti-hGLUT9b antibody. (**C**) HEK-293 cells overexpressing hGLUT9a stained with a rabbit anti-hGLUT9b antibody. (**D**) HEK-293 cells overexpressing hGLUT9b stained with a rabbit anti-hGLUT9a antibody.

Cells **2022**, *11*, 633. https://doi.org/10.3390/cells11040633 www.mdpi.com/journal/cells

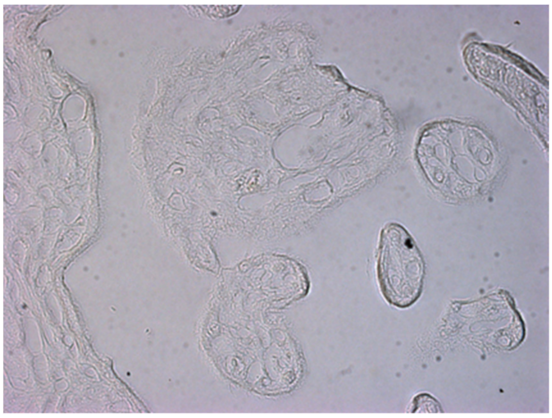

Supplementary Figure S2. Negative control of GLUT9-specific antibodies with term placentae. The negative control that was performed in the absence of the primary antibody revealed no detectable signal.

References:

1. Kimura, T.; Takahashi, M.; Yan, K.; Sakurai, H. Expression of SLC2A9 isoforms in the kidney and their localization in polarized epithelial cells. PLoS ONE 2014, 9, e84996.
